# Reduce, Reuse, Recycle, Run ! : 4 Rs to improve cardiac health in advanced age

**DOI:** 10.18632/aging.204415

**Published:** 2022-12-01

**Authors:** Jae Min Cho, Rajeshwary Ghosh, Sohom Mookherjee, Sihem Boudina, J. David Symons

**Affiliations:** 1 Department of Nutrition and Integrative Physiology, University of Utah, Salt Lake City, UT 84112, USA; 2 Division of Endocrinology, Metabolism and Diabetes, and Program in Molecular Medicine, University of Utah, Salt Lake City, UT 84112, USA

**Keywords:** autophagy, exercise training, cardiac function, heart

## Abstract

During the aging process damaged/dysfunctional proteins and organelles accumulate and contribute to organ dysfunction. Luckily, there is a conserved intracellular process to reuse and recycle these dysregulated cellular components termed macroautophagy (autophagy). Unfortunately, strong evidence indicates autophagy is compromised with aging, protein quality control is jeopardized, and resultant proteotoxicity can contribute significantly to age-associated organ dysfunction. Are there interventions that can re-establish autophagic flux that is otherwise impaired with aging? With particular regard to the heart, here we review evidence that caloric-restriction, the polyamine spermidine, and the mTOR inhibitor rapamycin, even when initiated late-in-life, restore cardiomyocyte autophagy to an extent that lessens age-associated cardiac dysfunction. Cho et al. provide a physiological intervention to this list i.e., regular physical exercise initiated late-in-life boosts cardiomyocyte autophagic flux and rejuvenates cardiac function in male mice. While this study provides strong evidence for a mechanism whereby heightened physical activity can lead to improved heart health in the context of aging, (i) only male mice were studied; (ii) the intensity of exercise-training might not be suitable for all; and (iii) mice with aging-associated comorbidities were not investigated. Nonetheless, Cho et al. provide robust evidence that a low-cost and simple behavioral intervention initiated late-in-life improves cardiomyocyte autophagic flux and rejuvenates cardiac function.

## INTRODUCTION

In North America life-expectancy was ~ 69 y in 1950, is ~ 79 y in 2022, and is projected to be ~ 89 y in 2100. Aging is an important risk factor for cardiac disease in both sexes and it cannot be modified [1]. For a multiplicity of reasons, and in response to internal and external sources of motivation, some individuals resolve to improve their heart health at an advanced age. Can this be done? If so, what is (are) the mechanism(s) responsible? The answer to the first question is YES! Insight concerning the second question is more nuanced, but studies using murine models of cardiac aging provide evidence that cardiomyocyte macroautophagy (referred to as autophagy) might be an efficacious target for intervention [2–5]. Autophagy is a process whereby damaged/dysfunctional proteins and organelles are targeted for degradation/recycling by the lysosome. If the recycling is not completed appropriately, defective proteins and organelles accumulate to an extent that precipitates oxidative stress and organ dysfunction [6]. In one of the first studies to provide strong proof of concept in the heart, adult mice with desmin-related cardiomyopathy characterized by the accumulation of misfolded proteins were crossed with mice allowing for cardiomyocyte-selective autophagy-related gene (Atg) 7 hyperactivation. Autophagic flux was greater and cardiac performance improved in these cardiomyopathic “gain of autophagy” mice [7].

In the following sections we review evidence that age-associated cardiac dysfunction can be Reduced by boosting cardiomyocyte autophagy (i.e., the ability to Reuse and Recycle damaged/dysfunctional proteins) via spermidine, rapamycin, and caloric-restriction. In addition, we highlight a new report indicating that a physiological intervention i.e., Running, rejuvenates cardiomyocyte autophagic flux to an extent that lessens age-associated cardiac dysfunction.

### Late-in-life interventions can improve cardiac autophagy

**Figure 1:**
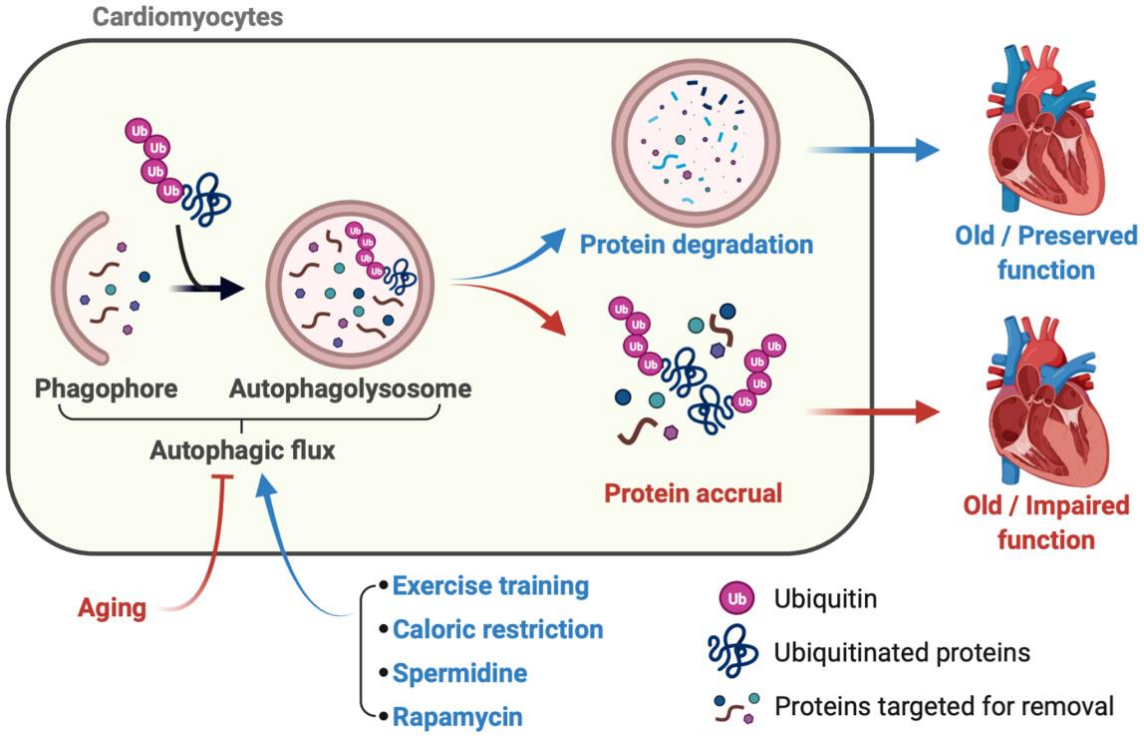
**Late-in-life exercise training boosts autophagic flux to an extent that rejuvenates cardiac function.** Accrual of ubiquitinated proteins that is, in part, secondary to impaired autophagic flux occurs over time and contributes to age-associated myocardial dysfunction (red lines and arrows). In mice, lifestyle (e.g., caloric restriction), nutraceutical (e.g., spermidine ingestion), and pharmacological (e.g., rapamycin-treatment) interventions initiated late-in-life boost cardiac autophagy and preserve myocardial function (blue lines and arrows). Cho et al. demonstrate that a physiological intervention (i.e., exercise-training), even when initiated late-in-life, improves autophagic flux, clears ubiquitinated proteins, reduces oxidant stress, and enhances cardiac function vs. results obtained from age-matched mice that did not train. These data provide the first evidence that habitual physical exercise, even when initiated late-in-life, is a viable adjunct “therapy” to improve/maintain myocardial performance during the inevitable process of aging.

While genetic manipulations cannot be used clinically to upregulate autophagy in conditions associated with cardiac aging at present, benefits from inducing this protein degradation pathway late-in-life via nutraceutical (e.g., spermidine), lifestyle (e.g., caloric restriction) and pharmacological (e.g. rapamycin) maneuvers have been demonstrated [3–5] (Figure 1). For example, cardiac hypertrophy was attenuated and diastolic function was preserved in mice that consumed spermidine-supplemented water from 18–24 months of age. The autophagy-boosting effect of this polyamine shown originally in flies and yeast was confirmed in the myocardium, and cardiac-selective autophagy-related gene (Atg) 5 deficient animals were refractory to this intervention, substantiating that autophagy is necessary for spermidine-evoked benefits to be observed [3]. Sheng et al. fed one group of mice standard chow ad libitum, whereas another cohort consumed 40% fewer calories from the same diet, from 19–22 months of age [4]. AMPK is phosphorylated and activated by nutrient-deprivation to an extent that increases expression of autophagy-associated genes and this was confirmed in myocardium from caloric-restricted vs. ad-libitum fed mice [4]. Age-associated cardiac fibrosis, left-ventricular hypertrophy, and compromised ejection fraction were less severe in mice that ingested caloric-restricted diet even when this intervention was initiated later in life. While inhibiting mammalian target of rapamycin complex 1 (mTORC1) signaling extends lifespan in organisms from worms to mice, Flynn et al. first reported the cardiovascular benefits of this intervention in the context of aging [5]. Mice treated with rapamycin from 24–27 months of age had repressed pro-inflammatory signaling in the myocardium, less left-ventricular hypertrophy, and preserved systolic function vs. age-matched controls. Although confirmation that rapamycin induced reductions in cardiac mTORC1 signaling was provided, evidence for upregulated cardiac autophagy *per se* was not presented in that study. While each of these approaches to boost autophagy in the context of aging attenuated indexes of cardiac dysfunction [3–5], it is unknown if the benefits associated positively with improved autophagic flux and protein clearance in the myocardium because neither of these endpoints was assessed. Furthermore, because off-target influences of spermidine may exist, caloric-restriction requires tremendous discipline, and rapamycin disrupts peripheral glucose homeostasis, doubt may be cast concerning the translational relevance of each of these interventions to stimulate cardiac autophagy and improve myocardial function in humans.

### Late-in-life exercise training improves cardiomyocyte autophagic flux

An additional lifestyle intervention with potential to improve autophagy, clear damaged proteins, and beneficially influence the aging-associated decline in cardiac function is dynamic exercise. He et al. first reported that an acute bout of treadmill-running activates autophagy in hearts from adult mice [8]. The Robbins laboratory group leveraged this finding to reveal that preamyloid oligomer, a toxic component in many protein misfolding-based neurodegenerative diseases, was less in hearts from adult mice with access to voluntary wheel-running (VWR) vs. those without this opportunity, but autophagy was not assessed in that study [9]. Later, the authors demonstrated that VWR increases mRNA expression of autophagy indexes in adult mice with cardiomyopathy, and it is particularly notable that cardiac function appears identical between cardiomyopathic “gain of autophagy” mice and cardiomyopathic animals that completed VWR, suggesting that cardiac protection afforded by genetic overexpression of autophagy and VWR is similar [7].

Cho, et al. tested the hypothesis that a late-in-life physiological intervention i.e., exercise training, increases cardiomyocyte autophagy to an extent that associates positively with improved cardiac function [2]. As expected, older mice that did not train exhibited impaired autophagic flux, greater protein aggregate accrual, exaggerated lipid oxidation, cardiac dysfunction, and reduced exercise capacity vs. inactive adult mice. To investigate the influence of late-in-life exercise training, additional cohorts of older mice did or did not complete a 3-month progressive resistance treadmill-running program, the efficacy of which was verified functionally and biochemically. Each of the listed endpoints that was disrupted by aging improved in mice that trained vs. those that did not train from 24–27 months of age. Concerning mechanisms, at least one possibility is that age-associated reductions in cardiomyocyte autophagy-related gene (Atg) 3 mRNA and protein expression were re-established in older trained animals. To test cause and effect, however, additional studies are required in older mice with inducible, cardiomyocyte specific autophagy-related gene (Atg) 3 depletion to evaluate the hypothesis that these mice are refractory to the effects of chronic physical activity concerning cardiac autophagic flux. Regardless of the precise mechanism, Cho et al. provide the first evidence that a physiological intervention i.e., late-in-life exercise-training, rejuvenates autophagic flux to an extent that clears protein aggregates and attenuates aging-associated cardiac dysfunction (Figure 1). Of additional interest in this study, although autophagy-competent adult male mice completed progressive resistance treadmill-running that evoked an efficacious training effect, cardiac autophagic flux was not affected. This is important because overactivation of autophagy associates with cell senescence in fibroblasts [10, 11] and cancer cells [11, 12] that are otherwise autophagy-competent.

### Considerations and future directions

The report from Cho et al. is not without limitations. First, older male mice were studied and it is entirely possible that the impact of habitual physical activity on cardiomyocyte autophagy might be more robust in older females when the cardioprotective influence of estrogen is less. Second, 24-month old mice trained at 70% of their maximal exercise capacity, and it is unlikely that older, previously inactive humans would choose or be advised to initiate a regular program of physical activity at this intensity. In addition to these considerations, results provided by the authors raise questions that inspire future scientific inquiry. For example, Cho et al. evaluated older mice that were otherwise healthy, but aging often associates with comorbidities (e.g., obesity, diabetes, hypertension, ischemia) known to independently influence cardiac autophagy. It would be interesting to determine if initiating regular physical exercise late-in-life improves autophagic flux to an extent that lessens the severity of cardiac dysfunction in these populations. Further, although the authors findings strongly suggest that chronic physical activity rejuvenates aging-associated cardiac dysfunction, and that this is associated with improved cardiomyocyte autophagic flux, protection might also be realized in response to an acute cardiovascular event. For instance, Campos et al. reported that myocardial infarction (MI)-induced heart failure in adult Wistar rats associated with reduced cardiac autophagic flux when assessed 4 and 12-weeks after the initial insult. An additional cohort of MI animals that trained from 4–12 weeks displayed improved autophagic flux and better cardiac function that was lost upon acute autophagy inhibition [13]. Because aging is an important risk factor for MI-induced heart failure, it would be extremely interesting to know whether regular physical activity improves cardiac autophagic flux in older animals to an extent that: (i) lessens the impact of MI-induced heart failure; and/or (ii) improves the rate and extent of recovery from MI-induced heart failure, and these studies are ongoing. Importantly, because myriad beneficial off-target (from autophagy) effects are associated with exercise-training, it is requisite to determine whether older mice with inducible, cardiac selective loss of autophagy are refractory to the benefits of chronic physical activity and these studies are in progress.

## CONCLUSIONS

Findings from Cho et al. suggest that age-associated cardiac dysfunction can be re-established by Reducing (physical inactivity), Reusing (lysosomal degradation products e.g., amino acids for ATP synthesis), Recycling (damaged intracellular organelles via the lysosome and other protein degradation pathways), and Running (or increasing physical activity via any mode that can be enjoyed regularly and safely by the individual) (Figure 1).

## References

[1] Virani SS, Alonso A, Aparicio HJ, Benjamin EJ, Bittencourt MS, Callaway CW, Carson AP, Chamberlain AM, Cheng S, Delling FN, Elkind MSV, Evenson KR, Ferguson JF, et al, and American Heart Association Council on Epidemiology and Prevention Statistics Committee and Stroke Statistics Subcommittee. Heart Disease and Stroke Statistics-2021 Update: A Report From the American Heart Association. Circulation. 2021; 143:e254–743. 10.1161/CIR.0000000000000950 33501848 PMC13036842

[2] Cho JM, Park SK, Ghosh R, Ly K, Ramous C, Thompson L, Hansen M, Mattera MSL, Pires KM, Ferhat M, Mookherjee S, Whitehead KJ, Carter K, et al. Late-in-life treadmill training rejuvenates autophagy, protein aggregate clearance, and function in mouse hearts. Aging Cell. 2021; 20:e13467. 10.1111/acel.13467 34554626 PMC8520717

[3] Eisenberg T, Abdellatif M, Schroeder S, Primessnig U, Stekovic S, Pendl T, Harger A, Schipke J, Zimmermann A, Schmidt A, Tong M, Ruckenstuhl C, Dammbrueck C, et al. Cardioprotection and lifespan extension by the natural polyamine spermidine. Nat Med. 2016; 22:1428–38. 10.1038/nm.4222 27841876 PMC5806691

[4] Sheng Y, Lv S, Huang M, Lv Y, Yu J, Liu J, Tang T, Qi H, Di W, Ding G. Opposing effects on cardiac function by calorie restriction in different-aged mice. Aging Cell. 2017; 16:1155–67. 10.1111/acel.12652 28799249 PMC5595678

[5] Flynn JM, O'Leary MN, Zambataro CA, Academia EC, Presley MP, Garrett BJ, Zykovich A, Mooney SD, Strong R, Rosen CJ, Kapahi P, Nelson MD, Kennedy BK, Melov S. Late-life rapamycin treatment reverses age-related heart dysfunction. Aging Cell. 2013; 12:851–62. 10.1111/acel.12109 23734717 PMC4098908

[6] Ghosh R, Vinod V, Symons JD, Boudina S. Protein and Mitochondria Quality Control Mechanisms and Cardiac Aging. Cells. 2020; 9:933. 10.3390/cells9040933 32290135 PMC7226975

[7] Bhuiyan MS, Pattison JS, Osinska H, James J, Gulick J, McLendon PM, Hill JA, Sadoshima J, Robbins J. Enhanced autophagy ameliorates cardiac proteinopathy. J Clin Invest. 2013; 123:5284–97. 10.1172/JCI70877 24177425 PMC3859422

[8] He C, Bassik MC, Moresi V, Sun K, Wei Y, Zou Z, An Z, Loh J, Fisher J, Sun Q, Korsmeyer S, Packer M, May HI, et al. Exercise-induced BCL2-regulated autophagy is required for muscle glucose homeostasis. Nature. 2012; 481:511–5. 10.1038/nature10758 22258505 PMC3518436

[9] Maloyan A, Gulick J, Glabe CG, Kayed R, Robbins J. Exercise reverses preamyloid oligomer and prolongs survival in alphaB-crystallin-based desmin-related cardiomyopathy. Proc Natl Acad Sci U S A. 2007; 104:5995–6000. 10.1073/pnas.0609202104 17389375 PMC1851605

[10] Young AR, Narita M, Ferreira M, Kirschner K, Sadaie M, Darot JF, Tavaré S, Arakawa S, Shimizu S, Watt FM, Narita M. Autophagy mediates the mitotic senescence transition. Genes Dev. 2009; 23:798–803. 10.1101/gad.519709 19279323 PMC2666340

[11] Capparelli C, Chiavarina B, Whitaker-Menezes D, Pestell TG, Pestell RG, Hulit J, Andò S, Howell A, Martinez-Outschoorn UE, Sotgia F, Lisanti MP. CDK inhibitors (p16/p19/p21) induce senescence and autophagy in cancer-associated fibroblasts, "fueling" tumor growth via paracrine interactions, without an increase in neo-angiogenesis. Cell Cycle. 2012; 11:3599–610. 10.4161/cc.21884 22935696 PMC3478311

[12] Huang YH, Yang PM, Chuah QY, Lee YJ, Hsieh YF, Peng CW, Chiu SJ. Autophagy promotes radiation-induced senescence but inhibits bystander effects in human breast cancer cells. Autophagy. 2014; 10:1212–28. 10.4161/auto.28772 24813621 PMC4203548

[13] Campos JC, Queliconi BB, Bozi LHM, Bechara LRG, Dourado PMM, Andres AM, Jannig PR, Gomes KMS, Zambelli VO, Rocha-Resende C, Guatimosim S, Brum PC, Mochly-Rosen D, et al. Exercise reestablishes autophagic flux and mitochondrial quality control in heart failure. Autophagy. 2017; 13:1304–17. 10.1080/15548627.2017.1325062 28598232 PMC5584854

